# Comparative Self-Evaluation of Patient Education Practice: A Study of Novice and Experienced Physiotherapists

**DOI:** 10.3390/healthcare13030260

**Published:** 2025-01-28

**Authors:** Vedrana Grbavac, Mladenka Naletilić, Josip Šimić, Roma Forbes

**Affiliations:** 1 Department of Physical Medicine and Rehabilitation, University Clinical Hospital Mostar, Ulica Kralja Tvrtka bb, 88000 Mostar, Bosnia and Herzegovina; mladenka.naletilic@fzs.sum.ba; 2 Faculty of Health Studies, University in Mostar, Zrinski Frankopan 34, 88000 Mostar, Bosnia and Herzegovina; josip.simic@fzs.sum.ba; 3 School of Health and Rehabilitation Sciences, The University of Queensland, Brisbane 4072, Australia; r.forbes2@uq.edu.au

**Keywords:** physiotherapy, training, teaching, experience

## Abstract

**Background:** Patient education is a key aspect of physical therapy practice; however, the differences in how experienced and novice physiotherapists perceive and apply patient education practice remain underexplored. Understanding these differences influences training approaches and improves physical therapy practice quality. This research aims to determine the difference in self-reported patient education practice between experienced and novice physiotherapists. **Methods:** A previously published online survey instrument was used to collect data from physiotherapists employed in public health institutions in Bosnia and Herzegovina. The survey questions included demographic characteristics and questions about approaches to patient education, perceived importance, and factors contributing to skills development. Participants were recruited in two groups: experienced physiotherapists with work experience ≥ 11 years (n = 139) and novice physiotherapists with work experience ≤ 5 years (n = 45). Descriptive statistics, such as numbers and percentages, were used to summarize participant responses. **Results:** Experienced physiotherapists more frequently provided advice on posture, movement correction, daily activity strategies, and pacing while addressing patient concerns and exploring perceptions (*p* < 0.05). In contrast, novice physiotherapists placed significantly greater value on continuing education courses, considering them an important factor in developing patient education skills (*p* < 0.05). **Conclusions:** Experienced physiotherapists prioritize patient education focusing on posture, movement, and self-care strategies compared to novice physiotherapists. However, novice physiotherapists place a higher importance role on continuous education. Identifying these differences may help tailor training and mentorship to improve physiotherapy practice, ensuring better patient outcomes.

## 1. Introduction

Patient education enables individuals to make informed decisions about their health-related behavior [1]. This includes teaching and encouraging patients to remain motivated to adhere to treatment recommendations through patient-centered educational strategies [2,3]. Patient-centered care is an individualized, holistic, relationship-centered approach that empowers patient outcomes, including in physiotherapy settings [4,5]. By empowering patients to active participation, physiotherapists can foster more effective and sustainable health behaviors and outcomes [6,7].

An early study by May and colleagues found that nearly all physical therapists (99%) considered patient education a key skill and 98% reported incorporating individual patient education into their practice [8] spending approximately 30% of therapy time on this aspect of their practices. Further research has shown that physiotherapists often use educational methods based on adult learning principles to enhance patients’ self-efficacy [9]. Through patient education, the physiotherapist encourages self-awareness and teaches and builds a collaborative relationship, which aligns with adult learning theories [10]. Despite the importance of patient education, physiotherapists reportedly struggle to integrate health promotion and stress reduction education into their routine practice and explaining the cause of a patient’s symptoms remains a challenge. In teaching others, including patients, experience and expertise play a key role in enabling the approach to the needs of the individual and the effective transfer of information [11].

Novice clinicians tend to rely on knowledge based on factual rules, whereas experts draw on their experience and practical [12]. Studies have found that novice therapists spend less time undertaking patient histories and more time undertaking a physical examination compared to experts [13] where expert clinicians consistently prioritize history-taking data as critical to the diagnostic reasoning process [14,15]. Experienced clinicians tend to approach patient care with a stronger patient-centered mindset, emphasizing shared decision-making and promoting patient empowerment [16,17]. However, limited research has addressed how novice physiotherapists approach patient education compared to experienced colleagues [18]. The education of patients in physiotherapy in Bosnia and Herzegovina varies, there is a lack of standardization and systematic documentation, which makes a complete review difficult; therefore, an analysis is needed to identify the existing practice in physiotherapy.

Early research has also shown that in interactions with patients, expert physiotherapists tend to provide more explanations and information during history taking, build their questions on patients’ responses, engage in more social interactions, and demonstrate higher communication skills than novice physiotherapists [19,20].

Experienced physiotherapists apply tacit knowledge (acquired through practice) in patient education, which allows them to quickly perform complex tasks, focus on key elements such as palpation and non-verbal communication, and work more efficiently compared to standard algorithms learned in school. This skill is developed through practical experiences and becomes automatic over time [21]. More recently, research in Australia has shown that experienced physiotherapists report greater use of self-management education, greater use of patient-centered content, greater use of seeking patient understanding, and fewer barriers to patient education than their novice colleagues, highlighting the need for further training and support [18].

Novice clinicians cite barriers to effective patient education [22], and even when instructed, often struggle with disorganized early-career transition programs [23]. These concerns extend to physiotherapy students. Holmes’s [24] findings suggest that physiotherapy students may not be fully aware of how their behaviors and beliefs may affect patients and that their preferences may be more aligned with a medical model (biological aspect) than with the biopsychosocial approach (biological, psychological, and social) [25]. These findings highlight the need to support students in the development of patient education during training.

Although it is often assumed that patient education skills, behaviors, and practices develop with experience, a literature review reveals a lack of research that specifically examines differences based on experience. A better understanding of the limitations faced by novice clinicians may be useful to improve the education of novice physiotherapists and students. By participating in continuing education and on-the-job training [26], physiotherapists acquire and improve new skills, with on-the-job training and practical experience playing a key role in developing educational skills. This study aims to explore how these factors may contribute to the differences observed between novice and experienced physical therapists [27].

In BiH, until now, physiotherapy research has been conducted by focusing on individual institutions, regions, or specific aspects of rehabilitation (certain diagnoses or therapies); therefore, there is a need for extensive research that would cover all hospitals where physical therapy is carried out, to obtain a clearer picture of patient education in physiotherapy. With this research, we aimed to investigate the differences in the self-assessment of patient education by experienced and novice physiotherapists, that is, how they perceive the importance of educational content, the methods they use, and the factors contributing to developing patient education skills. These findings may advance clinical practice by highlighting the importance of patient education and its integration into routine care. Also, they can serve as a basis for the standardization of educational elements in health policies and contribute to guidelines for the training of novice physiotherapists in the development of teaching, communication, and cooperation skills with patients.

## 2. Materials and Methods

### 2.1. Study Design

A cross-sectional study was conducted to assess patient education practices and perceptions of physiotherapists. The researchers requested ethical approval from all hospitals with physical medicine and rehabilitation clinics. Approval to participate in the study was sought from all hospitals with physical medicine and rehabilitation clinics in BiH. Three of the four hospitals obtained ethical approval to conduct the study (KBC Mostar, KBC Tuzla, and the Institute for Physical Medicine and Rehabilitation “Dr. Miroslav Zotović” in BiH).

After obtaining ethical approval, the researcher presented the study to physiotherapists working in the mentioned and introduced potential participants to the purpose of the research, the data collection procedure, and the method of protecting anonymity. The goal was to test physiotherapists with experience in patient education. A total of 212 physiotherapists were invited to participate, of whom 184 (87%) consented and were included in the analysis. Participation was voluntary, and participants provided written consent before completing the study. Participants were also given the choice of completing the survey electronically. Still, they did not use it (respondents did not utilize the electronic survey option, likely due to potential barriers, such as limited access to technology or physiotherapists’ preference for a paper survey format). Physiotherapists who were unqualified or not directly involved in patient care, such as teachers or administrative staff, were excluded from the study. The respondents returned the completed and enveloped surveys to the researcher. Data collection was conducted between June and August 2024. and collection lasted one week in each institution

### 2.2. Survey Method

A previously published [11] survey developed by Forbes and colleagues was requested for use in this study. The survey was selected because of its specific focus on assessing, and perceptions of physiotherapists in patient education. The anonymous survey focused on six key elements relevant to physiotherapy and patient education practices: the physiotherapy context, time, educational content, structure, and factors influencing skill development (such as a question about the methods of patient education or barriers). The survey included nine demographic questions, two multiple-choice questions about the duration of education, and six sets of closed-ended questions using a five-point Likert scale in matrix form (Cronbach alpha reliability coefficients ranged from 0.703 to 0.892). Participants assessed the frequency and importance of educational activities, as well as their agreement with factors that influence the development of skills necessary for effective patient education.

The translation into Croatian followed forward and backward translation procedures in line with recommended guidelines [28]. Two bilingual physiotherapists familiar with the terminology independently performed the initial forward translation into Croatian and agreed on a single version. Two independent bilingual translators, not involved in the earlier stages, then completed the backward translation into English. The survey was culturally adapted for relevance to the BiH healthcare context by adjusting terminology, removing irrelevant references, and resolving inconsistencies through focus groups until consensus was reached on the final version. The survey was pilot-tested with 15 physiotherapists to ensure clarity and consensus on the final version (available in Supplementary Materials).

### 2.3. Selection and Sample

The sample size was determined based on data from the Institute of Public Health of Bosnia and Herzegovina. The sample size was calculated using an online tool with a confidence level of 95%, a margin of error of 5%, a population proportion of 80%, and the population size of physiotherapists working in the hospital system. A total of 212 physiotherapists were invited to participate.

Earlier research has employed different approaches to characterize ’experienced’ health professionals, with criteria including at least ten years of work experience [16,18] completion of postgraduate education, or having practical expertise in several areas of the profession [23].

The criteria for defining a ’novice’ healthcare practitioner vary, with some studies setting the threshold at two or four years of experience [11]. For this study, novices were defined as having ≤5 years of practice, while experienced practitioners were those with ≥11 years. The group with 6–10 years of experience was not selected for analysis because it represents a transitional phase between beginners and highly experienced practitioners. These definitions ensured that the subgroups were large enough for meaningful comparisons. Participants are physiotherapists from hospital physical medicine and rehabilitation clinics from BiH with experience in educating inpatients and outpatients.

### 2.4. Data Analysis

Quantitative data were entered into a Microsoft Excel spreadsheet and checked for missing or incomplete responses. Only responses with more than 80% of the data completed were included in the analysis. For responses with less than 20% missing data, the mean imputation method within each variable was used, ensuring the accuracy and validity of the analysis without loss of data. The statistical analyses were conducted using Microsoft Excel and SPSS version 20.0. The Shapiro–Wilk test was applied to assess the normality of the data distribution. Numbers and percentages (n and %) were used to identify qualitative data, where the chi-square test was performed to compare categorical data, which could contain two or more groups. Continuous variables were described by their mean and standard deviation (SD), and compared using independent samples *t*-test while the Mann–Whitney U test was employed for ordinal data. Effect sizes were calculated using Cohen’s d (independent sample *t*-test), and r (Mann–Whitney u test) which was calculated by dividing the Z score by the square root of the subject number (Z/sqrt(N)). All tests were bilateral, with a *p*-value of *p* < 0.05 considered statistically significant.

### 2.5. Ethical Approval

Ethical approval for this research was obtained from the Ethics Committee of the University Clinical Hospital Mostar (No. 91/24), University Clinical Center Tuzla (02-09/2-49-1/24), and Institute for Physical Medicine and Rehabilitation “Dr. Miroslav Zotović” (116-31-5671-2/24). To avoid a potential influence on the perception of voluntariness, the survey was conducted in each clinic for one week. The time frame ensured that the pressure on potential respondents was reduced, and the completion and submission of the questionnaire did not involve personal contact (with colleagues, supervisors, or researchers) to ensure anonymity. These measures contributed to the creation of conditions in which the participants could freely decide to participate in the research. The return of the questionnaire was organized in closed envelopes that were not marked with identification data, which made it impossible to identify the participants.

## 3. Results

A total of 184 responses were gathered, yielding a response rate of 87%. Nearly half of the physiotherapists, or 47.1%, have between 11 and 20 years of work experience, whereas the smallest groups consisted of those with 1 to 2 years (6.3%) or less than 1 year of experience (6.3%). The remaining data sets (n = 24) with 6–10 years of work experience were excluded from the analysis.

### 3.1. Differences in Self-Assessment of Educational Content Use Between Experienced Physiotherapists and Novices

Before determining potential differences in self-assessment of educational content use between experienced and novice physiotherapists, the basic descriptive parameters of sociodemographic variables for the two groups were outlined (Table 1).

In both groups, there are slightly more female than male respondents (51.1% compared to 48.9% among novices and 66.2% compared to 33.8% among experienced). Most novices, as expected (68.9%), are aged between 20 and 29 years. The majority of novices (53.3%) and experienced physiotherapists (41%) cited musculoskeletal as their professional interest. Novices and experienced physiotherapists do not differ in their educational level (*p* < 0.05).

### 3.2. Differences in the Amount of Patient Education Concerning Work Experience

The majority of physiotherapists (44.7%) reported spending more than 15 min educating patients during the initial consultation, while 37.5% spent a similar amount of time during follow-up consultations. A small percentage of physiotherapists (2.4%) spent only 1–3 min on patient education during the first consultation, with slightly more (4.3%) performing the same in regular appointments. Results of the Mann–Whitney U test (Table 2) revealed no statistically significant differences in the duration of education during either initial or follow-up appointments between physiotherapists with varying levels of work experience (*p* > 0.05).

### 3.3. Differences in Activities Used During Education Based on Physiotherapists’ Work Experience

The independent samples *t*-test (Table 3) revealed that experienced physiotherapists, compared to novices, were significantly more likely to provide advice or instruction on posture and movement correction, inquire about the patient’s concerns, offer strategies for performing activities of daily living, advise on the pace of daily activities, and explore the patient’s ideas and perceptions (*p* < 0.05).

### 3.4. Differences in the Ratings of the Importance of Patient Education Content Based on Work Experience

Most questions assessing the perceived importance of educational content did not show significant differences in the ratings between novice and experienced physiotherapists. However, experienced physiotherapists rated advising or teaching correct posture and movement, as well as teaching self-care strategies, and providing information about the patient’s prognosis, as significantly more important than novices (*p* < 0.05), as shown in Table 4.

### 3.5. Differences in the Evaluation of Patient Education Effectiveness Between Novice and Experienced Physiotherapists

Using independent samples *t*-test, no significant differences were found in the evaluation of patient education effectiveness based on work experience (*p* > 0.05, Table 5).

### 3.6. Differences in the Perception of Necessary Factors for the Development of Patient Education Skills Between Novice and Experienced Physiotherapists

An analysis of the attitudes of novice and experienced physiotherapists revealed significant differences regarding the value of continuing education courses (independent sample *t*-test). Novice physiotherapists rated continuing education as a significantly more important factor for developing patient education skills compared to their counterparts with more than 11 years of experience (*p* < 0.05, Table 6).

## 4. Discussion

This study compared novice and experienced physiotherapists’ self-reported use and perception of patient education. The results show that experienced physical therapists emphasize the importance of addressing patient concerns and perceptions, teaching proper posture or movement, teaching self-management strategies, and emphasizing practical experience when compared to their novice colleagues. Physiotherapists reported that patient education is an integral part of their work, with nearly half stating that they spend more than 15 min educating patients during initial consultations, while over one-third reported performing so during follow-up consultations. The results of the survey study indicate that experienced physiotherapists prioritize counseling, teaching, and empowering patients to manage their health and explore the patient’s ideas and perceptions when compared to novice physiotherapists (*p* < 0.05). Novice physiotherapists considered prior training and experience before studying physiotherapy to be the least important, whereas experienced physiotherapists placed greater value on practical experience, viewing courses further education as the least impactful for skill development.

Similarly, qualitative research involving novice and experienced healthcare professionals in cardiovascular settings suggests that experienced professionals are better at tailoring education to patients’ specific needs and circumstances [29]. This patient-centered approach considers the patient’s desire for information and shapes education from the patient’s perspective [30,31], which can be the basis for improving educational practice. No significant differences were found between novice and experienced physiotherapists in their perception of the importance of most educational content. However, experienced physiotherapists placed more emphasis on counseling, teaching, and educating patients compared to less experienced colleagues. These findings are consistent with previous qualitative research, which shows that experienced physical therapists actively promote patient self-management and work to improve patient self-efficacy [32]. Research with physicians [33] and patients [34] indicates that more experienced practitioners tend to have a deeper understanding of the impact of self-management skills on patient care and health outcomes related to novice practitioners. Differences in practice and perceptions of patient education between novice and experienced physiotherapists are likely influenced by years of experience and the presence and content of on-the-job training, which plays a key role in helping novices acquire knowledge and skills as they transition to experienced therapists [35]. These results contribute to the stated need for education and training of physical therapists, especially inpatient self-control management strategies. Research by Svavarsdottir et al. indicates similar levels of patient education ability between novice and experienced therapists [29]. One main reason that may explain these findings is that physical therapists receive minimal training in patient education skills [36], and continuing professional development activities are rarely conducted in this area [37]. Most courses intended for physiotherapists are aimed at more technical clinical skills and often do not meet the educational needs [37,38]. Evaluation is considered the final phase of patient-centered education, despite its importance, evaluation often receives limited focus in training health professionals [39]. The results show that no significant differences were found in the evaluation of patient education effectiveness based on experience, with the majority of respondents determining that they very often or always seek feedback from the patient. Feedback is crucial in education as it allows physiotherapists (novices and experienced) to better understand and develop skills, and increase their effectiveness in clinical work with patients [40]. Similar findings were reported in Australian research [18], although our results also highlight a notable absence of written instructions and advice for patients, which may be attributed to differing regulations and approaches in the health system.

The highest-rated factors contributing to developing patient education skills were ‘personal experience with patients’, ‘interaction with colleagues’, and ‘continuing education courses’. The literature highlights that early-career clinicians are often still in the process of independent learning and require significant support in the workplace [41]. Taking on the role of a patient educator adds extra responsibilities, such as providing guidance, feedback, and assessments [42]. These duties, combined with the well-documented challenges of patient education, may contribute to feelings of apprehension, or even anxiety, among novice clinicians [43,44,45]. While this study did not directly examine anxiety levels, previous research has shown that less experienced clinicians tend to feel more anxious in managing their professional roles compared to their more experienced counterparts [46], which may be relevant to the context of our findings. Novice physiotherapists prioritize continuing education to develop patient education skills, while considering prior education the least important, whereas experienced physiotherapists value practical experience more and place less importance on additional courses. Emphasizing the importance of continuous education by beginners, reflects their strong motivation to acquire new knowledge and skills, which positions them as potential drivers of innovation in clinical practice. Experienced physiotherapists may prioritize practical experience as they have accumulated tacit knowledge through years of clinical experience. Their confidence in managing patient interactions may stem from repeated exposure to diverse cases, enabling them to refine their ability to tailor education to individual patient needs. This evolution aligns with Kolb’s experiential learning theory, which emphasizes learning through reflection on hands-on experiences [47]. Additionally, experienced practitioners’ familiarity with common clinical challenges allows them to focus on practical problem-solving rather than seeking external validation through structured learning. By participating in continuous education and training in the workplace, physiotherapists acquire and develop new skills, while training and practical experience play a key role in the development of educational competencies [48]. However, in BiH, training at the workplace is not systematically organized, the mentoring program for beginners has not been developed, and the specialization program for physiotherapists is not adequately harmonized. The study highlights the differences between novice and experienced physiotherapists but does not address the educational background or systems that might influence the development of these differences.

The findings of this study, while grounded in the specific context of BiH, have significant implications for physiotherapy education globally. Similarly to trends observed in other nations, the emphasis novice physiotherapists place on continuing education aligns with the growing recognition of the need for lifelong learning in healthcare professions. This study highlights the importance of adapting educational programs to not only address localized needs but also to align with international standards, ensuring graduates are equipped for cross-border practice.

Integrating the perspectives of novice and experienced therapists: These results highlight the key advantages and challenges associated with the different phases of the professional development of physiotherapists through the role of an educator; however, it underscore the advantages of novices and their potential contribution. Novices emphasize a strong motivation for continuous education, often creating an equal relationship with patients, and approach empathically. Their openness to innovation can improve the patient experience and drive change in clinical practice. In contrast, experienced therapists offer a wealth of practical knowledge and intuition gained over years of work. However, their tendency to rely on set protocols, and established practice [21] can limit adaptation to individual patient needs. The synergy between novice and experienced therapists offers significant advantages for clinical practice and patient education. Novices can bring innovative techniques and the latest knowledge, while experienced therapists can provide mentorship and impart practical skills [49]. This collaboration creates a dynamic environment where both parties learn from each other, resulting in continuous professional development, better clinical outcomes, and patient-centered care.

Future Directions: Future research should include longitudinal studies that investigate the progress of novice therapists through their career development as educators, as well as intervention studies that investigate the effectiveness of educational approaches such as mentoring and simulations. We recommend similar comparative studies from different countries, especially those facing similar health challenges, to uncover common challenges in physiotherapist education, as well as successful approaches that could be applied in different contexts. Comparison with international practice could lead to the development of globally relevant educational strategies that would support the development of therapists in different health systems.

Limitations: A major impact of this study stems from the extensive experience most participants had in patient education in physiotherapy, particularly as most had experience in various educational settings. Although the inclusion of experienced physiotherapists enriched the findings of the study, the smaller number of novices may limit the perspective and lead to the neglect of the specific challenges faced by new physiotherapy educators. Future studies should ensure that novices are better represented to obtain a more comprehensive picture. The study’s main limitation was basing the results on the personal and professional opinions of the physiotherapists, rather than on their actual work. This approach was deliberately chosen due to the lack of robust descriptions of novice and expert educators in physiotherapy. This study’s reliance on self-reported data introduces potential biases, including social desirability bias, where participants may have reported behaviors and attitudes they believed were expected rather than their actual practices. Future studies should consider direct observation of physiotherapist-patient interactions or utilize observational tools to validate self-reported findings.

## 5. Conclusions

This study provides valuable insight into physiotherapist practice and perceptions regarding patient education. Experienced physiotherapists in this study prioritized teaching proper posture, movement, and self-care strategies, while novice physiotherapists emphasized the importance of continuing education. Given the critical role of self-management education in increasing patient satisfaction and health outcomes in physiotherapy, as well as the established relationship between patient-centered care and patient outcomes, the lower utilization of key educational content reported by novice therapists could impact patient care. Consequently, future research should focus on the factors that make differences between novice and experienced therapists and the importance of gaining experience, self-efficacy, and practice skills. This study could serve as the first step in a research program aimed at improving physiotherapist training curricula.

## Figures and Tables

**Table 1:** Distribution of Novice and Experienced Physiotherapists Regarding Basic Sociodemographic Characteristics (N = 184).

Variable		Work Experience		
		Novices N (%)	Experienced N (%)	*p* ^a^
Gender	Male	22 (48.9)	47 (33.8)	0.069
	Female	23 (51.1)	92 (66.2)	
Age	20–29	31 (68.9)	2 (1.5)	**<0.001**
	30–39	12 (26.7)	64 (46)	
	40–49	2 (4.4)	48 (34.5)	
	50–59	0	18 (13)	
	60+	0	7 (5)	
Work Experience	<1 year	13 (28.9)	0	**<0.001**
	1–2 years	7 (15.5)	0	
	3–5 years	25 (55.6)	0	
	6–10 years	0	0	
	11–20 years	0	98 (70.5)	
	20+ years	0	41 (29.5)	
Educational Level	High School Diploma	4 (8.9)	17 (12.2)	0.173
	Bachelor’s Degree	33 (73.3)	107 (77)	
	Master’s Degree	8 (17.8)	11 (10.8)	
		M = 15.089 ± 1.24	M = 14.785 ± 1.193	
		t ^b^ = 1.464, *p* = 0.145, Cohen’s d = 0.25		
Professional Interest	Musculoskeletal System	24 (53.3)	57 (41)	0.326
	Cardiorespiratory System	0	11 (7.9)	
	Neurology	10 (22.2)	32 (23)	
	Pediatrics	3 (6.7)	14 (10.1)	
	Sports	4 (8.9)	10 (7.2)	
	Women’s Health	0	6 (4.3)	
	Elderly Care	0	1 (0.7)	
	Other	4 (8.9)	8 (5.8)	

^a^ *p*-values for the chi-square test were provided, ^b^ results of independent samples *t*-test for examining differences in the educational level between novices and experienced (bold *p*-value indicates statistical significance).

**Table 2:** Distribution of physiotherapists about the duration of patient education and differences concerning work experience (N = 184).

Variable	Work Experience	Novices N (N = 45)		Experienced (N = 139)					
		N (%)	Mean Rank	N (%)	Mean Rank	Mann–Whitney U Test	Z-Value	*p*-Value	Effect Size (r)
Duration of education during the first visit	1–3 min	0	92.16	5 (3.6)	91.95	3098	−0.024	0.981	0.001
	4–6 min	7 (15.6)		8 (5.8)					
	6–10 min	4 (8.9)		33 (23.9)					
	11–15 min	15 (33.3)		29 (21)					
	>15 min	19 (42.2)		63 (45.7)					
Duration of education during regular sessions	1–3 min	4 (8.9)	90.2	5 (3.6)	93.24	3024	−0.347	0.728	0.256
	4–6 min	8 (17.8)		20 (14.4)					
	6–10 min	9 (20)		43 (30.9)					
	11–15 min	7 (15.5)		20 (14.4)					
	>15 min	17 (37.8)		51 (36.7)					

**Table 3:** Differences in the Activities Used During Patient Education Based on Work Experience (N = 184).

	Novices (N = 45)	Experienced (N = 139)			
Activity	M ± Sd ^a^	M ± Sd	t ^b^	*p*-Value	Effect Size (Cohen’s d)
Providing information about the patient’s condition or diagnosis	3.622 ± 0.86	3.755 ± 1.135	−0.722	0.471	0.132
Providing verbal or written exercise instructions	4.022 ± 0.917	4.166 ± 0.914	−0.913	0.362	0.157
Providing advice or instruction on posture or movement correction	4.222 ± 0.765	4.439 ± 0.703	−1.757	0.081	0.295
Advice or instruction on self-correction strategies	4 ± 0.826	4.317 ± 0.817	−2.254	0.025	0.386
Asking about the patient’s concerns	3.356 ± 0.743	3.799 ± 0.957	−2.839	0.005	0.517
Providing information about the patient’s prognosis	3.156 ± 1.107	3.086 ± 1.067	0.375	0.708	0.064
Advice and strategies for performing activities of daily living	3.889 ± 0.775	4.259 ± 0.966	−2.337	0.021	0.423
Exploring the patient’s ideas and perceptions	2.978 ± 0.988	3.353 ± 0.97	−2.243	0.026	0.383
Advice on the patient’s activity pace	3.644 ± 0.83	4.022 ± 0.867	−2.562	0.011	0.445
Advice on social support	2.978 ± 0.892	3.101 ± 0.995	−0.738	0.461	0.13
Consultation about stress, emotional, or psychological problems	2.956 ± 0.976	3.144 ± 1.114	−1.015	0.311	0.18
Promoting health	3.6 ± 0.809	3.871 ± 0.931	−1.746	0.082	0.311
Advice or instruction on addressing health-related problems	3.467 ± 0.815	3.748 ± 0.844	−1.962	0.051	0.339
Explaining pain neurophysiological mind–body	3.222 ± 0.823	3.518 ± 0.958	−1.86	0.065	0.331
Advice on using assistive equipment or devices	3.89 ± 0.935	3.86 ± 1.018	0.191	0.848	0.031

^a^ values are expressed on a Likert scale: min = 1 (“never”), max = 5 (“always”), ^b^ independent samples *t*-test.

**Table 4:** Differences in the Ratings of the Importance of Patient Education Content Based on Physiotherapists’ Work Experience (N = 184).

Variable	Experience Level		M ± Sd ^a^	t ^b^	*p*-Value	Effect Size (Cohen’s d)
Providing information about the patient’s condition or diagnosis		Novice ^c^	3.91 ± 0.874	−1.312	0.191	0.2354
		Experienced ^d^	4.13 ± 0.999			
Providing verbal or written instructions for performing basic exercises		Novice	4.42 ± 0.621	−0.293	0.770	0.056
		Experienced	4.46 ± 0.801			
Advising or teaching correct posture and movement		Novice	4.36 ± 0.679	−2.872	0.006	0.516
		Experienced	4.68 ± 0.555			
Advising or teaching self-care strategies		Novice	4.18 ± 0.684	−2.039	0.043	0.344
		Experienced	4.42 ± 0.712			
Asking the patient about health concerns and discussing them		Novice	3.56 ± 0.785	−1.151	0.251	0.202
		Experienced	3.73 ± 0.891			
Providing information about the patient’s prognosis		Novice	3.27 ± 0.78	−2.039	0.044	0.319
		Experienced	3.57 ± 1.077			
Advice or strategies for performing daily life activities		Novice	4.16 ± 0.721	−1.734	0.085	0.312
		Experienced	4.4 ± 0.814			
Exploring patients’ ideas and perceptions		Novice	3.42 ± 0.932	−1.707	0.090	0.293
		Experienced	3.69 ± 0.908			
Advising or teaching about the patient’s activity pace		Novice	3.98 ± 0.913	−0.16	0.873	0.023
		Experienced	4 ± 0.808			
Advice on social support		Novice	3.47 ± 0.882	−0.613	0.540	0.101
		Experienced	3.56 ± 0.902			
Advising on stress, emotional, or psychosocial problems		Novice	3.56 ± 0.765	−0.639	0.524	0.114
		Experienced	3.66 ± 0.975			
General health promotion		Novice	3.93 ± 0.704	0.168	0.867	0.026
		Experienced	3.91 ± 0.842			
Advising or teaching strategies for solving problems related to diagnosis		Novice	3.88 ± 0.697	−0.726	0.469	0.143
		Experienced	3.99 ± 0.834			
Explaining the neurophysiology of pain/mind–body, description of pain		Novice	3.67 ± 0.837	−0.011	0.991	0.011
		Experienced	3.68 ± 0.987			
Advice on using assistive devices or equipment		Novice	4.05 ± 0.872	0.114	0.910	0.023
		Experienced	4.03 ± 0.9			

^a^ values are expressed on a Likert scale: min = 1 (“never”), max = 5 (“always”), ^b^ independent samples *t*-test, ^c^ N = 45, ^d^ N = 139.

**Table 5:** Differences in the Evaluation of Patient Education Effectiveness Based on Work Experience (N = 184).

Activity	Group	M ± Sd ^a^	t ^b^	*p*-Value	Effect Size (Cohen’s d)
Ask the patient to repeat the content in their own words	Novice ^c^	3.31 ± 1.221	0.597	0.551	0.099
	Experienced ^d^	3.19 ± 1.207			
Ask the patient to demonstrate	Novice	4.33 ± 0.739	0.399	0.690	0.066
	Experienced	4.28 ± 0.78			
Interpret patient’s signals of understanding	Novice	4 ± 0.853	0.164	0.870	0.025
	Experienced	3.98 ± 0.737			
Objective measures or standards	Novice	3.69 ± 1.019	−0.454	0.650	0.072
	Experienced	3.76 ± 0.921			
Ask family members or caregivers.	Novice	3.18 ± 1.072	0.662	0.509	0.118
	Experienced	3.05 ± 1.138			
Analyze patient tasks via video Activity	Novice	2.2 ± 1.217	0.751	0.454	0.127
	Experienced	2.05 ± 1.144			

^a^ values are expressed on a Likert scale: min = 1 (“never”), max = 5 (“always”), ^b^ independent samples *t*-test, ^c^ N = 45, ^d^ N = 139.

**Table 6:** Differences in the perception of necessary factors for the development of patient education skills between novice and experienced physiotherapists (N = 184).

Activity	Group	M ± Sd ^a^	t ^b^	*p*-Value	Effect Size (Cohen’s d)
Training and/or experience before school or study	Novice ^c^	3.53 ± 0.842	0.137	0.891	0.022
	Experienced ^d^	3.51 ± 0.995			
Academic/university study of physiotherapy	Novice	4.09 ± 0.668	1.761	0.080	0.329
	Experienced	3.82 ± 0.95			
Continuing education courses	Novice	3.76 ± 0.802	2.648	0.009	0.482
	Experienced	3.33 ± 0.973			
Postgraduate studies	Novice	4.2 ± 0.661	0.755	0.451	0.143
	Experienced	4.09 ± 0.867			
Professional services in the service	Novice	4.13 ± 0.726	0.437	0.663	0.069
	Experienced	4.08 ± 0.723			
Interaction with colleagues	Novice	4.31 ± 0.701	1.548	0.123	0.275
	Experienced	4.1 ± 0.819			
Personal experience with patients	Novice	4.36 ± 0.645	−0.368	0.713	0.063
	Experienced	4.4 ± 0.633			

^a^ values are expressed on a Likert scale: min = 1 (“never”), max = 5 (“always”), ^b^ independent samples *t*-test, ^c^ N = 45, ^d^ N = 139.

## Data Availability

The data presented in this study are available on request from the corresponding author. The data are not publicly available due to privacy reasons.

## Supplementary Materials

The following supporting information can be downloaded at: https://www.mdpi.com/article/10.3390/healthcare13030260/s1.
