# Supplementary material for: Comparative Self-Evaluation of Patient Education Practice: A Study of Novice and Experienced Physiotherapists
Source: Healthcare (Basel). 2025 Jan 28;13(3):260. doi: 10.3390/healthcare13030260 (PMC11817888; doi:10.3390/healthcare13030260)
Supplement: Supplementary file 1 [file healthcare-13-00260-s001.zip › healthcare-3374716-supplementary.pdf]

The table shows the key differences between experienced and novice physiotherapists concerning patient education.

Category	Experienced Physiotherapists	Beginner Physiotherapists
**Approach to Patient Education**	Patient-centered, focusing on individual needs and patient perceptions.	Rely on standardized guidelines and theoretical knowledge, with less adjustment to the patient's specific needs.
**Methods of Patient Education**	Verbal and written instructions, exercise demonstrations, exploration of ideas, and patient perceptions.	Rely more on verbal instructions and basic information, with fewer demonstrations and less exploration of perceptions.
**Content of Patient Education**	Focus on posture, movement correction, daily activity strategies, activity timing, and self-care.	Emphasis on basic information about the condition and diagnosis, with less focus on self-care strategies and activity adjustments.
**Time Spent on Education**	More than half of therapists spend around 15 minutes during initial and regular consultations.	More than half of therapists spend around 15 minutes during consultations.
**Importance of Continuous Education**	Less emphasis on formal courses; focus on practical experience and collaboration with colleagues.	Consider continuous educational courses key to developing patient education skills.
**Communication with Patients**	Actively explore the patient's concerns, ideas, and perceptions.	Focus less on patient perceptions, relying on generalized instructions.
**Self-management Topics**	Education on self-managing pain, maintaining proper posture, and activities of daily life ($p < 0.05$).	Less frequently cover self-management topics; focus on diagnostic information and technical aspects of exercises.
**Learning and Development Sources**	Personal experience with patients and interaction with colleagues are key factors in developing education skills.	Emphasis on formal education and courses as primary sources of skill development ($p < 0.05$).
**Evaluation of Patient Education**	Always or very often ask the patient to repeat the content and demonstrate what they've learned, interpreting signs of understanding.	Always or very often ask the patient to repeat the content, but ask less frequently for signs of learning or demonstrations.
